# An independently validated nomogram for isocitrate dehydrogenase-wild-type glioblastoma patient survival

**DOI:** 10.1093/noajnl/vdz007

**Published:** 2019-05-30

**Authors:** Haley Gittleman, Gino Cioffi, Pranathi Chunduru, Annette M Molinaro, Mitchel S Berger, Andrew E Sloan, Jill S Barnholtz-Sloan

**Affiliations:** 1 Department of Population and Quantitative Health Sciences, Case Western Reserve University School of Medicine, Cleveland, Ohio; 2 Case Comprehensive Cancer Center, Case Western Reserve University School of Medicine, Cleveland, Ohio; 3 Department of Neurological Surgery, University of California San Francisco, San Francisco, California; 4 Department of Neurological Surgery, University Hospitals of Cleveland and Case Western University School of Medicine, Cleveland, Ohio; 5 Seidman Cancer Center, University Hospitals of Cleveland, Cleveland, Ohio

**Keywords:** glioblastoma, IDH-wild-type, nomogram, survival

## Abstract

**Background:**

In 2016, the World Health Organization reclassified the definition of glioblastoma (GBM), dividing these tumors into isocitrate dehydrogenase (IDH)-wild-type and IDH-mutant GBM, where the vast majority of GBMs are IDH-wild-type. Nomograms are useful tools for individualized estimation of survival. This study aimed to develop and independently validate a nomogram for IDH-wild-type patients with newly diagnosed GBM.

**Methods:**

Data were obtained from newly diagnosed GBM patients from the Ohio Brain Tumor Study (OBTS) and the University of California San Francisco (UCSF) for diagnosis years 2007–2017 with the following variables: age at diagnosis, sex, extent of resection, concurrent radiation/temozolomide (TMZ) status, Karnofsky Performance Status (KPS), O^6^-methylguanine-DNA methyltransferase (MGMT) methylation status, and IDH mutation status. Survival was assessed using Cox proportional hazards regression, random survival forests, and recursive partitioning analysis, with adjustment for known prognostic factors. The models were developed using the OBTS data and independently validated using the UCSF data. Models were internally validated using 10-fold cross-validation and externally validated by plotting calibration curves.

**Results:**

A final nomogram was validated for IDH-wild-type newly diagnosed GBM. Factors that increased the probability of survival included younger age at diagnosis, female sex, having gross total resection, having concurrent radiation/TMZ, having a high KPS, and having *MGMT* methylation.

**Conclusions:**

A nomogram that calculates individualized survival probabilities for IDH-wild-type patients with newly diagnosed GBM could be useful to physicians for counseling patients regarding treatment decisions and optimizing therapeutic approaches. Free software for implementing this nomogram is provided: https://gcioffi.shinyapps.io/Nomogram_For_IDH_Wildtype_GBM_H_Gittleman/.

**Key points**

1. A nomogram for IDH-wild-type glioblastoma patients has been developed and externally validated.
2. Free software for implementing this nomogram is provided allowing for the ease of use by practicing healthcare providers.

**Importance of the Study**

Glioblastoma (GBM) is the most common malignant brain tumor. In 2016, the World Health Organization reclassified the definition of GBM, dividing these tumors into isocitrate dehydrogenase (IDH)-wild-type and IDH-mutant GBM, where the vast majority of GBMs are IDH-wild-type. A nomogram accounts for several prognostic factors and is an easily accessible tool for physicians to use on behalf of their patients for predicting survival, developing individualized cancer prognosis, and deciding the interval for follow-up and/or imaging. A nomogram for assessing survival estimates for IDH-wild-type patients with GBM has been developed and independently validated. To facilitate clinical use of this nomogram, free software for its implementation is provided (https://gcioffi.shinyapps.io/Nomogram_For_IDH_Wildtype_GBM_H_Gittleman/). The nomogram provides an individualized estimate of survival rather than a group estimate. This tool can be useful to patients and healthcare providers for counseling patients and their families regarding treatment decisions, follow-up, and prognosis.

Glioblastoma (GBM) is the most common malignant brain and other central nervous system tumor, comprising 14.7% of all primary brain tumors, 47.7% of all malignant brain tumors, and 56.6% of all gliomas in the United States.^1–14^ Patients with GBM have poor prognosis, with a 5-year relative survival rate of 5.6% and median overall survival of 12–15 months.^1–8,15,16^ Prognostic factors include age, sex, extent of surgical resection, treatment, Karnofsky performance status (KPS), and race, as well as certain biomarkers.^1–3,9,15,17^ These biomarkers include methylation status of the gene promoter for O^6^-methylguanine-DNA methyltransferase (MGMT) and isocitrate dehydrogenase enzyme 1/2 (IDH1/2) mutation.^1,2,4,18–20^ In 2016, the World Health Organization (WHO) reclassified the definition of GBM, dividing these tumors into IDH-wild-type and IDH-mutant GBM.^18^ IDH mutations are rare in GBM, accounting for less than 10% of patients with GBM.^4,19^ The current standard of care for GBM consists of maximal safe surgical resection followed by radiotherapy with concurrent temozolomide (TMZ) chemotherapy, sometimes followed by adjuvant TMZ.^1,2,5,9–11,16,17,19,21^

Nomograms are accessible tools for physicians to use on behalf of their patients for predicting survival, developing an individualized cancer prognosis valuable for treatment decision-making, and deciding the interval for follow-up and/or imaging.^22^ Two nomograms have been developed previously for GBM. The first used data from a European Organisation for Research and Treatment of Cancer-National Cancer Institute of Canada (EORTC-NCIC) clinical trial (EORTC 26981/22981-NCIC); however, this nomogram was only internally validated.^23^ The second, developed by our team, used data from 2 independent, nonoverlapping NRG Oncology Radiation Therapy Oncology Group clinical trials, 0525 and 0825.^22^ Although this GBM nomogram was both internally and externally validated, it did not include some important prognostic factors, such as whether or not the patient received concurrent radiation/TMZ and IDH mutation status.

Because of the 2016 WHO redefinition of GBM, the purpose of this study was to develop and independently validate a nomogram for the estimation of individualized survival probabilities for newly diagnosed IDH-wild-type GBM patients that would be easy to use through an online interface. The final nomogram was created using data from the Ohio Brain Tumor Study (OBTS) and externally validated using data from the University of California San Francisco (UCSF).

## Materials and Methods

### Data Collection and Study Population

This study was approved by the University Hospitals and UCSF Institutional Review Boards. De-identified data were provided by the OBTS and the UCSF for the diagnosis years 2007–2017 on newly diagnosed GBM patients who were at least 18 years of age at diagnosis. GBM was defined using ICD-O-3 codes 9440/3, 9441/3, and 9442/3. The OBTS data initially included 218 newly diagnosed GBM patients, whereas the UCSF data initially included 128 newly diagnosed GBM patients. The following variables were obtained for each patient: age at diagnosis (continuous), sex (male or female), race (white, black, or other), extent of surgical resection (biopsy, subtotal resection, gross total resection; gross total resection defined as >98% of the enhancing region of the tumor resected when comparing pre- vs postoperative MRI), KPS (10–100 in tens) recorded postsurgery, concurrent radiation/TMZ (yes or no), IDH1 mutation (yes or no), *MGMT* methylation (yes or no), survival/follow-up time in months (continuous), and survival status (alive or dead). Because of the small sample of patients with low values for KPS, this variable was dichotomized into at least 70 and less than 70. Because the UCSF data only included patients who had subtotal or gross total surgical resection, patients who had biopsy only were dropped from the OBTS data, leaving a total of 200 patients left in the OBTS dataset. Missing data were imputed using multivariate imputation by chained equations using the R package “mice.”^24^ The raw, unimputed data for the 2 datasets are displayed in Supplementary Table 1. After data were imputed, IDH1-mutant patients (21 in OBTS and 6 in UCSF) were removed. The final OBTS dataset had a total of 179 patients, and the final UCSF dataset had a total of 122 patients, both sets only including patients with IDH-wild-type tumors.

All GBMs were histologically confirmed. *IDH* testing was conducted via immunohistochemistry and reviewed at each participating center by an expert neuropathologist. *MGMT* methylation testing was performed using a standard pyrosequencing approach where purified genomic DNA was treated with sodium bisulfite to convert unmethylated cytosines into uracil. After conversion, a targeted region was amplified by polymerase chain reaction. Methylation of 5 CpG islands in an *MGMT* promoter region was analyzed by pyrosequencing using Qiagen PyroMark Q96ID (Qiagen, Germany). Percentage of methylation of each CpG island was analyzed and a percent mean of *MGMT* methylation is reported. Percent mean methylation 10% or greater was considered positive.

### Statistical Analyses

Descriptive statistics were used to assess any differences in patient characteristics and prognostic factors between the 2 datasets using *t*-tests for continuous variables and chi-square tests for categorical variables in the unimputed and imputed datasets. Using the “survival” package in R^25^, overall survival, as well as adjusted survival by age at diagnosis and KPS, were calculated for each of the 2 datasets using the Kaplan–Meier method, and differences were assessed using the log-rank test. Adjusting for age at diagnosis, sex, extent of surgical resection, concurrent radiation/TMZ, KPS, and *MGMT* methylation status, overall survival was assessed using Cox proportional hazards (CPH) regression (“survival” package),^25^ random survival forests (RSF) (“randomForestSRC” package),^26^ and recursive partitioning analysis (RPA) (“rpart” package).^27^ In CPH, the proportional hazards assumption was verified using the methodology by Grambsch and Therneau.^28^ In RSF, an ensemble tree method for analyzing right-censored survival data was used to generate 1000 trees, and all possible split points for each variable were evaluated to find the optimal split solution. In RPA, a full recursive classification tree was generated and the least important splits were removed recursively to obtain the final subclassification tree with the minimal model deviance.

The models were trained using the OBTS data and independently, externally validated using the UCSF data. Models were also internally validated using 10-fold cross-validation, and individual predicted 12-, 18-, and 24-month survival probabilities were generated to measure predictive accuracy compared with the observed survival as “ground truth.” The predictive accuracies for overall survival of the 3 statistical approaches (CPH, RSF, and RPA) were calculated using the concordance index, which ranges from 0.5 (completely random prediction) to 1 (perfect prediction) and is equivalent to the area under the receiver operating characteristic curve for censored data.^29^ A final nomogram was developed using the method with the greatest predictive accuracy for individualized estimation of survival. Calibration of the final model was visually examined by assigning all patients into quintiles of the nomogram-predicted 12-month survival probabilities and plotting the mean nomogram predicted 12-month survival probability against the Kaplan–Meier estimated 12-month survival for each quintile using the “rms” R package.^30^ All analyses were performed using R version 3.5.2.^31^*P*-values less than .05 were considered to be statistically significant.

## Results

### Patient Characteristics

Patient demographics for the imputed, IDH-wild-type OBTS patients (*N* = 179) and the UCSF patients (*N* = 122) are presented in Table 1. There were several statistically significant differences between the training (OBTS) and validation (UCSF) datasets. More patients (59.8%) in the OBTS set had gross total surgical resection, whereas more patients (62.3%) in the UCSF set had subtotal surgical resection (*P* < .001). More patients (79.5%) in the UCSF set had concurrent radiation/TMZ compared with those in the OBTS set (65.9%;*P* = .015). Overall, 67.0% of OBTS patients had a KPS greater or equal to 70 compared with 86.9% of UCSF patients (*P* < .001). Finally, a greater proportion of patients died in the OBTS dataset (91.1%) compared with the UCSF dataset (74.6%;*P* < .001). Patients in the OBTS dataset were also older at diagnosis (mean age = 63.02 years) than patients in the UCSF dataset (mean age = 60.90 years), though this was not significantly different (*P* = .104).

**Table 1.** Isocitrate Dehydrogenase-Wild-Type Newly Diagnosed Primary Glioblastoma Patient Characteristics Using Imputed Data; the Ohio Brain Tumor Study (OBTS) and the University of California San Francisco (UCSF), 2007–2017

	OBTS (training set) (*N* = 179)	UCSF (validation set) (*N* = 122)	*P*-value
Age at diagnosis (mean (SD)) [Range]	63.02 (10.98) [29–88]	60.90 (11.28) [24–85]	.104
Sex (*N* (%))			.180
Male	119 (66.5)	71 (58.2)	
Female	60 (33.5)	51 (41.8)	
Surgery status (*N* (%))			<.001
Subtotal resection	72 (40.2)	76 (62.3)	
Gross total resection	107 (59.8)	46 (37.7)	
Concurrent radiation/TMZ (*N* (%))			.015
Yes	118 (65.9)	97 (79.5)	
No	61 (34.1)	25 (20.5)	
KPS (*N* (%))			<.001
<70	59 (33.0)	16 (13.1)	
≥70	120 (67.0)	106 (86.9)	
MGMT methylation (*N* (%))			.745
Yes	79 (44.1)	57 (46.7)	
No	100 (55.9)	65 (53.3)	
Follow-up months (median [IQR])	12.07 [5.71, 19.75]	13.59 [7.79, 19.19]	.128
Survival status (*N* (%))			<.001
Alive	16 (8.9)	31 (25.4)	
Dead	163 (91.1)	91 (74.6)	

IQR = interquartile range; TMZ = temozolomide.

### Survival by the Kaplan–Meier Method and CPH

**Fig. 1:**
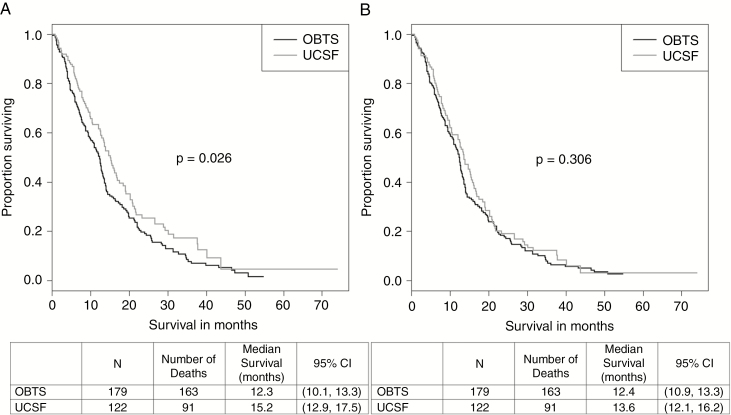
Kaplan–Meier survival curves for isocitrate dehydrogenase-wild-type newly diagnosed glioblastoma patients from the Ohio Brain Tumor Study and the University of California San Francisco, 2007–2017 (A) unadjusted and (B) adjusted for age at diagnosis and Karnofsky Performance Status.

Without adjusting for covariates, the Kaplan–Meier curve (Figure 1A) showed a significant difference (*P* = .026) between the OBTS and UCSF datasets, with the OBTS patients (median survival = 12.3 months; 95% confidence interval [CI]: 10.1–13.3 months) having worse survival than the UCSF patients (median survival = 15.2 months; 95% CI: 12.9–17.5 months). However, after adjusting for age at diagnosis and KPS (Figure 1B), this significant difference disappeared (*P* = .306). The median overall survival for the OBTS patients was 12.4 months (95% CI: 10.9–13.3 months), and the median overall survival for the UCSF patients was 13.6 months (95% CI: 12.1–16.2 months). Univariable and multivariable CPH regression results are displayed in Supplementary Table 2 and Table 2, respectively, for both the training set (OBTS) and the validation set (UCSF), displayed separately. In the multivariable analyses, younger age at diagnosis, having concurrent radiation/TMZ, having a KPS of 70 or greater, and having *MGMT* methylation were significantly associated with better survival outcomes (all *P*s < .05) in both datasets. Being male was significantly associated with worse survival (*P* = .011) in the OBTS dataset, but not in the UCSF dataset (*P* = .402). There was no significant difference between having gross total surgical resection versus subtotal surgical resection in either of the datasets. The data did not violate the proportional hazards assumption of the CPH model.

**Table 2.** Multivariable Cox Proportional Hazards Models for Isocitrate Dehydrogenase-Wild-Type Newly Diagnosed Glioblastoma Patients From the Ohio Brain Tumor Study (OBTS) and the University of California San Francisco (UCSF), 2007–2017

	OBTS (Training; *N* = 179)			UCSF (Validation; *N* = 122)		
Factor	HR	95% CI	*P*-value	HR	95% CI	*P*-value
Age	1.018	(1.002–1.034)	.026	1.025	(1.005–1.045)	.016
Sex (male vs female)	1.596	(1.114–2.285)	.011	1.206	(0.778–1.870)	.402
Surgery (STR vs GTR)	1.084	(0.788–1.493)	.620	1.011	(0.634–1.611)	.965
Concurrent radiation/TMZ (yes vs. no)	0.244	(0.161–0.369)	<.001	0.504	(0.301–0.845)	.009
KPS (≥70 vs. <70)	0.351	(0.237–0.520)	<.001	0.308	(0.163–0.583)	<.001
MGMT methylation (yes vs. no)	0.579	(0.395–0.848)	.005	0.474	(0.299–0.764)	.002

CI = confidence interval; GTR = gross total resection; HR = hazard ratio; KPS = Karnofsky Performance Status; MGMT = O^6^-methylguanine-DNA methyltransferase; STR = subtotal resection; TMZ = temozolomide.

### Internal Cross-Validation

After 10-fold cross-validation was performed on the training set (OBTS), the concordance indices were computed for each statistical method for predicting survival at 3 time points: 12, 18, and 24 months (Table 3). For all 3 time points, the CPH analysis (12 , 18, 24 months: 0.756, 0.757, 0.759) outperformed RSF (12, 18, 24 months: 0.752, 0.740, 0.708) and RPA (12, 18, 24 months: 0.747, 0.747, 0.747). On the basis of these results, the multivariable CPH model was then independently validated using the UCSF dataset.

**Table 3.** Concordance Indices and Corresponding 95% Confidence Intervals for Cox Proportional Hazards (CPH), Random Survival Forests (RSF), and Recursive Partitioning Analysis (RPA) at 12, 18, and 24 Months for Isocitrate Dehydrogenase-Wild-Type Newly Diagnosed Glioblastoma Patients; the Ohio Brain Tumor Study (Training Set), 2007–2017

	CPH	RSF	RPA
12 Months	0.756 (0.719–0.793)	0.752 (0.715–0.788)	0.747 (0.710–0.784)
18 Months	0.757 (0.721–0.794)	0.740 (0.704–0.776)	0.747 (0.710–0.784)
24 Months	0.759 (0.722–0.795)	0.708 (0.667–0.749)	0.747 (0.710–0.784)

### Nomogram and Independent Validation

**Fig. 2:**
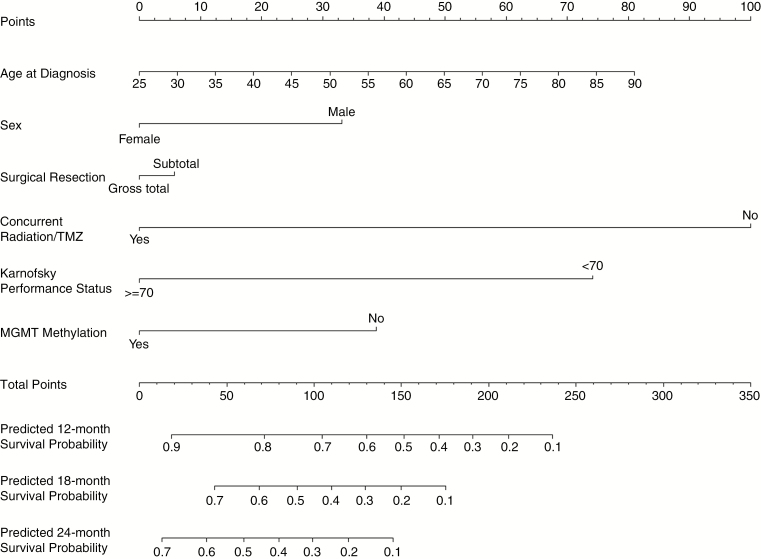
Validated nomogram for predicted 12-, 18-, and 24-month survival for newly diagnosed isocitrate dehydrogenase-wild-type glioblastoma patients; the Ohio Brain Tumor Study, 2007–2017.

**Fig. 3:**
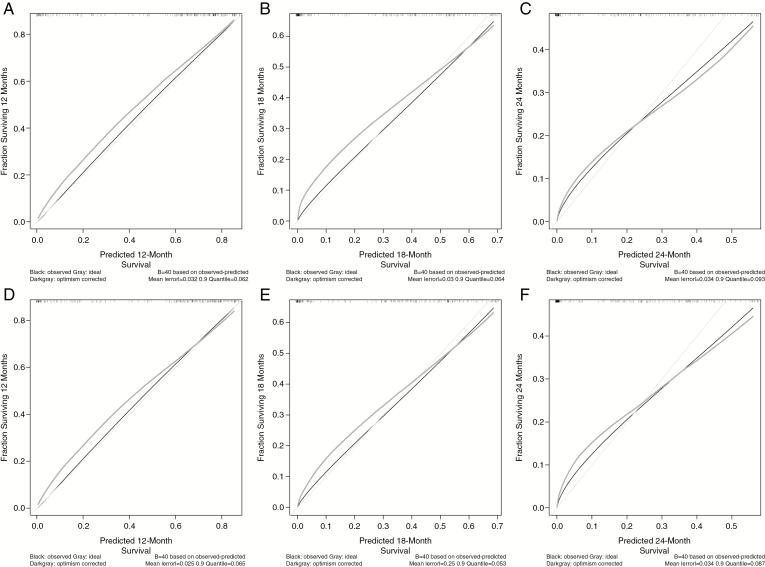
Calibration curves for training data (the Ohio Brain Tumor Study) for predicted (A) 12-, (B) 18-, and (C) 24-month survival and testing data (University of California San Francisco) for predicted (D) 12-, (E) 18-, and (F) 24-month survival for isocitrate dehydrogenase-wild-type newly diagnosed glioblastoma patients, 2007–2017.

The nomogram to estimate 12-, 18-, and 24-month survival probabilities was built using the training dataset (OBTS) and validated on the independent dataset (UCSF) using the CPH model (Figure 2). An online calculator for the final nomogram is available via an internet interface at https://gcioffi.shinyapps.io/Nomogram_For_IDH_Wildtype_GBM_H_Gittleman/. The final CPH model was well calibrated with a concordance index of 0.756 (95% CI: 0.719–0.793) in the training dataset. For each quintile group, the estimated versus observed 12-month survival probabilities intersected the 45° line, indicating that the predicted value approximated the observed value within a 95% CI (Supplementary Figure 1). Calibration curves were also drawn for the training dataset (OBTS) for predicted 12-, 18-, and 24-month survival, as well as for the independent validation dataset (UCSF) for a visual comparison. In Figure 3, the black line shows the observed survival rates, the gray line shows the ideal survival rates, and the dark gray line shows the optimism-corrected survival rates. The optimism-corrected (also known as bias-corrected or overfitting-corrected) line is produced using a bootstrap approach to estimate predicted and observed values based on a nonparametric smoother applied to a sequence of predicted values. For the predicted 12-month survival plots (Figure 3A and D), all 3 lines are closely aligned, demonstrating good calibration. For the predicted 18-month survival plots (Figure 3B and E), the observed and the ideal 45° lines are well aligned, although the optimism-corrected line strays somewhat from the others. For the predicted 24-month survival plots (Figures 3C and F), the observed and optimism-corrected lines are well-aligned, although both of these lines stray from the ideal 45° line, showing some overconfidence.

## Discussion

The goal of this study was to develop and validate, both internally and externally, an individual survival nomogram for patients with newly diagnosed IDH-wild-type GBM. The multivariable CPH regression model had the best fit, with high values for the concordance indices at 12, 18, and 24 -months (all concordance indices > 0.75). This CPH survival model adjusted for age at diagnosis, sex, surgical resection, concurrent radiation/TMZ, KPS, and *MGMT* methylation status, and was then independently, externally validated on the UCSF dataset.

Our previous nomogram on all newly diagnosed GBM patients regardless of IDH mutation status included age at diagnosis, sex, KPS, surgical resection, and *MGMT* methylation status.^22^ We built on this nomogram by subsetting to IDH-wild-type patients only, and adding other important prognostic variables such as concurrent radiation/TMZ.^1,2,5,9–11,16,17,19,21^ Even with these differences, the 2 nomograms share some similarities. For example, the difference between having gross total surgical resection and subtotal surgical resection, as well as the difference between having methylated versus unmethylated *MGMT*, earned approximately the same amount of points on both nomograms.^22^ The current nomogram found male sex to be a more severe marker of worse survival than the older nomogram.^22^ KPS cannot be compared because the current nomogram dichotomized this variable (≥70 compared with <70), whereas the older nomogram only included patients who had a KPS of 70 or higher.^22^ It is important to note that the older nomogram was developed using clinical trial data (training *N* = 799; validation *N* = 555)^22^ and therefore had a more robust sample size than that of the current study (training *N* = 179; validation *N* = 122).

IDH mutations define a distinct subset of GBM with a favorable outcome.^1,4,19,20^ These mutations are more common in lower grade gliomas such as grade II and grade III astrocytomas and oligodendrogliomas, than in GBM.^1,4,19^ Survival of GBM patients with such IDH-mutated tumors is more favorable than for nonmutated grade II astrocytoma, emphasizing the strong prognostic value of this marker.^19^ Because of the WHO 2016 reclassification of the definition of GBM into IDH-wild-type and IDH-mutant GBM,^18^ nomograms for GBM patients should be developed separately for these 2 unique entities. Because more than 90% of the mutations involve IDH1 rather than IDH2^1,19^, our subset of IDH-wild-type patients is defined by the IDH1 marker. Future work could include developing and validating a nomogram for IDH-mutant GBM patients.

There are several limitations to this study. First, the OBTS and UCSF datasets had several differences in their patient populations. The UCSF validation set had a younger, healthier patient population than that of OBTS. Treatment patterns varied as well, with more patients in the OBTS set having had gross total surgical resection and more patients in the UCSF set having had subtotal surgical resection, and more patients in the UCSF set having had concurrent radiation/TMZ than those in the OBTS set. Patients in the UCSF set had better overall survival compared with patients in the OBTS set; however, this difference disappeared after adjusting for age at diagnosis and KPS. Second, because the nomogram was trained from an Ohio population and validated on patients from California, both of which include world-class tertiary referral centers, the results may not be generalizable to patients treated at community hospitals. Third, although race was collected on patients in both datasets, nearly all of the patients were white (91.1% in the OBTS set and 94.2% in the UCSF set). Therefore, race was not found to be a significant factor and was dropped from the survival models, despite evidence for differences in survival by race in other studies.^1,4^ Fourth, because all the patients in the UCSF set had either subtotal or gross total surgical resection, biopsy-only patients were removed from the OBTS set before analysis. Thus, the nomogram may not be predictive for biopsy-only GBM patients. Fifth, although the OBTS set collected data on whether or not patients received adjuvant TMZ, the UCSF set did not collect this variable, so this variable was not used in the nomogram. Future work could include this adjuvant TMZ variable. Sixth, there was a small amount of missing data in some of the variables including concurrent radiation/TMZ, and a larger amount of missing data in KPS, *MGMT* methylation, and IDH1 mutation status. Although missing data were imputed using multivariate imputation by chained equations, creating 20 imputed datasets, which were pooled together into 1 final complete dataset, it is possible to have some level of inaccuracy in the imputed values. Because the missing values were derived from other clinical variables in the dataset, variables with missing data such as KPS and *MGMT* may be somewhat reliant on age or other clinical factors. Finally, although the internal and external calibration plots yielded nearly perfect calibration at 12 months and good calibration at 18 months, the observed and optimism-corrected lines deviated somewhat from the ideal line in both the internal and external validation at 24 months. This could be due to relatively small sample sizes in the training and validation datasets, which become smaller in 10-fold cross-validation. Caution should be used in the interpretation of the nomogram when analyzing patients with an unusual combination of clinical characteristics, as these patients may not be adequately represented in the training set from which the nomogram was derived.

A nomogram for obtaining survival estimates for IDH-wild-type patients with newly diagnosed GBM has been developed and independently validated. This tool provides an individualized estimate of survival, rather than a group estimate based on specific patient-level characteristics, which should be useful to patients and healthcare providers for counseling patients and their families regarding treatment decisions, follow-up, and prognosis. To facilitate the clinical use of this nomogram, free software for its implementation is provided (https://gcioffi.shinyapps.io/Nomogram_For_IDH_Wildtype_GBM_H_Gittleman/).

## Supplementary Material

vdz007_suppl_Supplementary_Figure_1

vdz007_suppl_Supplementary_Table_1

vdz007_suppl_Supplementary_Table_2

vdz007_suppl_Supplementary_Table_and_Figure_Legends
