# Supplementary figures and images for: An independently validated nomogram for isocitrate dehydrogenase-wild-type glioblastoma patient survival
Source: Neurooncol Adv. 2019 May 30;1(1):vdz007. doi: 10.1093/noajnl/vdz007 (PMC6777501; doi:10.1093/noajnl/vdz007)

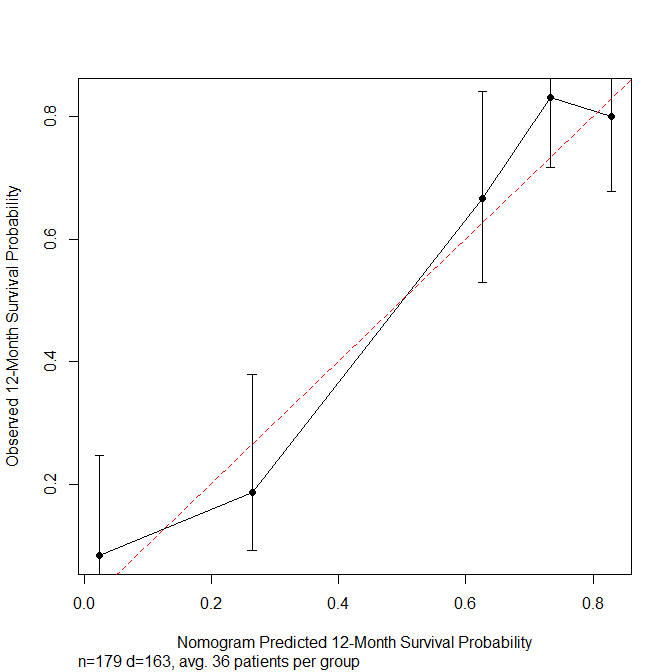

Supplement: vdz007_suppl_Supplementary_Figure_1 [file vdz007_suppl_supplementary_figure_1.png]
