# Supplementary material for: An independently validated nomogram for isocitrate dehydrogenase-wild-type glioblastoma patient survival
Source: Neurooncol Adv. 2019 May 30;1(1):vdz007. doi: 10.1093/noajnl/vdz007 (PMC6777501; doi:10.1093/noajnl/vdz007)
Supplement: vdz007_suppl_Supplementary_Table_1 [file vdz007_suppl_supplementary_table_1.docx]

**Supplementary Table 1. Newly diagnosed GBM patient characteristics using unimputed data; OBTS and UCSF, 2007-2017**

|  | **OBTS (N = 200)** | **UCSF (N = 128)** | **P-Value** |
| --- | --- | --- | --- |
| Age at Diagnosis (mean (sd)) | 62.19 (11.50) | 60.03 (12.09) | 0.106 |
| Sex (N (%)) | | | 0.272 |
| Male | 132 (66.0%) | 76 (59.4%) |  |
| Female | 68 (34.0%) | 52 (40.6%) |  |
| Surgery Status (N (%)) | | | <0.001 |
| Subtotal Resection | 81 (40.5%) | 78 (60.9%) |  |
| Gross Total Resection | 119 (59.5%) | 50 (39.1%) |  |
| Concurrent Radiation/TMZ (N (%)) | (MISSING  N = 16; 8.0%) |  | 0.035 |
| Yes | 124 (67.4%) | 101 (78.9%) |  |
| No | 60 (32.6%) | 27 (21.1%) |  |
| KPS (N (%)) | (MISSING  N = 56; 28.0%) | (MISSING  N = 12; 9.4%) | 0.003 |
| <70 | 43 (29.9%) | 16 (13.8%) |  |
| ≥70 | 101 (70.1%) | 100 (86.2%) |  |
| IDH1 Mutation (N (%)) | (MISSING  N = 87; 43.5%) |  | 0.300 |
| Yes | 10 (8.8%) | 6 (4.7%) |  |
| No | 103 (91.2%) | 122 (95.3%) |  |
| MGMT Methylation (N (%)) | (MISSING  N = 164; 82.0%) |  | 0.952 |
| Yes | 18 (50.0%) | 61 (47.7%) |  |
| No | 18 (50.0%) | 67 (52.3%) |  |
| Follow-up Months (median [IQR]) | 12.10  [5.77, 20.07] | 13.74  [7.64, 19.44] | 0.180 |
| Survival Status (N (%)) | | | <0.001 |
| Alive | 21 (10.5%) | 34 (26.6%) |  |
| Dead | 179 (89.5%) | 94 (73.4%) |  |
