# Supplementary material for: An independently validated nomogram for isocitrate dehydrogenase-wild-type glioblastoma patient survival
Source: Neurooncol Adv. 2019 May 30;1(1):vdz007. doi: 10.1093/noajnl/vdz007 (PMC6777501; doi:10.1093/noajnl/vdz007)
Supplement: vdz007_suppl_Supplementary_Table_2 [file vdz007_suppl_supplementary_table_2.docx]

**Supplementary Table 2. Univariable Cox proportional hazards models for IDH-wildtype newly diagnosed GBM patients from OBTS and UCSF, 2007-2017**

|  | **OBTS [Training] (N = 179)** | | | **UCSF [Validation] (N = 122)** | | |
| --- | --- | --- | --- | --- | --- | --- |
| **Factor** | **HR** | **95% CI** | **P-Value** | **HR** | **95% CI** | **P-Value** |
| Age | 1.022 | (1.008, 1.038) | 0.003 | 1.019 | (0.999, 1.040) | 0.066 |
| Sex (Male vs. Female) | 1.504 | (1.067, 2.120) | 0.020 | 1.236 | (0.808, 1.892) | 0.329 |
| Surgery (STR vs. GTR) | 1.178 | (0.862, 1.611) | 0.304 | 1.148 | (0.747, 1.763) | 0.529 |
| Concurrent Radiation/TMZ (Yes vs. No) | 0.152 | (0.105, 0.219) | <0.001 | 0.414 | (0.251, 0.683) | <0.001 |
| KPS (≥70 vs. <70) | 0.362 | (0.259, 0.505) | <0.001 | 0.393 | (0.221, 0.701) | 0.002 |
| MGMT Methylation (Yes vs. No) | 0.560 | (0.405, 0.775) | <0.001 | 0.522 | (0.340, 0.802) | 0.003 |
