# Supplementary material for: An independently validated nomogram for isocitrate dehydrogenase-wild-type glioblastoma patient survival
Source: Neurooncol Adv. 2019 May 30;1(1):vdz007. doi: 10.1093/noajnl/vdz007 (PMC6777501; doi:10.1093/noajnl/vdz007)
Supplement: vdz007_suppl_Supplementary_Table_and_Figure_Legends [file vdz007_suppl_supplementary_table_and_figure_legends.docx]

**Supplementary Table and Figure Legends**

Supplementary Table 1. Newly diagnosed GBM patient characteristics using unimputed data; OBTS and UCSF, 2007-2017

Supplementary Table 2. Univariable Cox proportional hazards models for IDH-wildtype newly diagnosed GBM patients from OBTS and UCSF, 2007-2017

Supplementary Figure 1. Calibration of the final model at 12 months for newly diagnosed IDH-wildtype GBM patients; OBTS (training set), 2007-2017
